# Duration of Purkinje cell complex spikes increases with their firing frequency

**DOI:** 10.3389/fncel.2015.00122

**Published:** 2015-04-13

**Authors:** Pascal Warnaar, Joao Couto, Mario Negrello, Marc Junker, Aleksandra Smilgin, Alla Ignashchenkova, Michele Giugliano, Peter Thier, Erik De Schutter

**Affiliations:** ^1^Theoretical Neurobiology and Neuroengineering Lab, Department of Biomedical Sciences, University of AntwerpWilrijk, Belgium; ^2^Department of Neuroscience, Erasmus MCRotterdam, Netherlands; ^3^Computational Neuroscience Unit, Okinawa Institute of Science and Technology, Onna-SonOkinawa, Japan; ^4^Department of Cognitive Neurology, Hertie Institute for Clinical Brain Research, University of TübingenTübingen, Germany; ^5^Physiology of Active Vision, Werner Reichardt Centre for Integrative Neuroscience, University of TübingenTübingen, Germany; ^6^Department of Computer Science, University of SheffieldSheffield, UK; ^7^Brain Mind Institute, Swiss Federal Institute of Technology LausanneLausanne, Switzerland

**Keywords:** Purkinje neuron, complex spike, monkey, waveform, saccades

## Abstract

Climbing fiber (CF) triggered complex spikes (CS) are massive depolarization bursts in the cerebellar Purkinje cell (PC), showing several high frequency spikelet components (±600 Hz). Since its early observations, the CS is known to vary in shape. In this study we describe CS waveforms, extracellularly recorded in awake primates (Macaca mulatta) performing saccades. Every PC analyzed showed a range of CS shapes with profoundly different duration and number of spikelets. The initial part of the CS was rather constant but the later part differed greatly, with a pronounced jitter of the last spikelets causing a large variation in total CS duration. Waveforms did not effect the following pause duration in the simple spike (SS) train, nor were SS firing rates predictive of the waveform shapes or* vice versa*. The waveforms did not differ between experimental conditions nor was there a preferred sequential order of CS shapes throughout the recordings. Instead, part of their variability, the timing jitter of the CS’s last spikelets, strongly correlated with interval length to the preceding CS: shorter CS intervals resulted in later appearance of the last spikelets in the CS burst, and* vice versa*. A similar phenomenon was observed in rat PCs recorded *in vitro* upon repeated extracellular stimulation of CFs at different frequencies in slice experiments. All together these results strongly suggest that the variability in the timing of the last spikelet is due to CS frequency dependent changes in PC excitability.

## Introduction

The Purkinje cell (PC) is the main point of converging pathways and the sole output neuron of the cerebellar cortex. Two strikingly different input pathways provide its excitatory input: (i) the massive convergence of granule cell axons onto a single PC’s distal dendrites; and (ii) the very strong connection by the climbing fiber (CF), originating in the Inferior Olive (IO) and branching over the PC dendrite proximal part. The granule cell input modulates simple spike (SS) firing, ranging up to 200 Hz. CF input triggers complex spikes (CS) at a remarkably low frequency (1 Hz). CSs show a massive calcium influx, during which multiple somatic Na^+^ spikes are fired. Following CSs the PC shows ±20 ms long pauses in the SS train (Bell and Grimm, 1969; Latham and Paul, 1971; McDevitt et al., 1982). These are attributed to both a depolarization related refractory period, resulting from Ca^2+^ activated-K^+^-hyperpolarizing currents (Edgerton and Reinhart, 2003), and to CF collateral triggered molecular layer interneuron inhibition (Szapiro and Barbour, 2007; Mathews et al., 2012). Interestingly, those pauses may be involved in the transfer of information downstream, which through concerted disinhibition of the cerebellar nuclei could trigger rebound bursts (De Schutter and Steuber, 2009; Maiz et al., 2012). CSs can induce long-term depression at parallel fiber synapses on PCs, which may be involved in cerebellar learning (Ito, 2001; Steuber et al., 2007). Despite decades of study and its essential roles in most cerebellar theories, evidence of their functional significance remains unclear. It has been suggested to represent an error signal in cerebellar learning (Ito, 2001; Kawato et al., 2011), to act as a stabilizing factor in motor learning (Catz et al., 2005), and to operate as a timing signal for temporal coordination (Llinás, 2009; Lefler et al., 2013). These conflicting roles ask for a closer look into the properties of CSs in behaving animals.

Early observations on CS waveforms reported shape variability (Eccles et al., 1966; Latham and Paul, 1971), but did not describe this variability systematically. It is known that the shape of a CS correlates with the intensity of intracellular current injections *in vitro* (Davie et al., 2008) and that the number of spikelets within a CS can be modulated by the number of spikes in a CF burst (Mathy et al., 2009). In rat cerebellar slices the pre-synaptic terminal of the CF-PC connection displays paired-pulse depression at physiological CS interval length ranges, resulting in a decreased number of spikelets in the second complex spike (Hashimoto and Kano, 1998). Furthermore the CF signal is modulated by the IO subthreshold oscillation amplitude (Bazzigaluppi et al., 2012) and could therefore act as a read out signal on the IO’s state and/or have a differentially instructive signal. In fact, a recent study reports that the CS duration and spikelet number correlate with the amount of learning in monkey PCs (Yang and Lisberger, 2014). Furthermore it has been shown that through the closed loop between PC, cerebellar nuclei and the IO, SS firing controls IO activity in parts that have efferents to the PCs were the SS originated. Control of the recurrent IO afferent input by SS activity was shown in both classical eye blink conditioning experiments (Rasmussen et al., 2014) and by optogenetic stimulation of the PC (Chaumont et al., 2013). This raises three questions: what is the variability of the CS shape in awake behaving animals? Is this variability mainly in spikelet numbers, or does it also occur in other features? Finally, is CS variability correlated with other features of the spike train that could provide insights into the mechanisms underlying CS variability? These questions are addressed in this paper by a systematic analysis of CS waveforms, observed in macaques during resting state and while performing a saccade fixation task.

## Material and Methods

All experimental procedures were performed in agreement with institutional, federal and European ethical guidelines and laws for animal experimentation.

All animal preparations and procedures fully complied with the National Institutes of Health Guide for the Care and Use of Laboratory Animals and were approved by the local animal care committee (Regierungspräsidium Tübingen, FG Tierschutz; Germany).

During the saccade task the monkeys were motivated to work by receiving a liquid reward (juice or water), while the intake of water was monitored according to the guidelines of the DPZ (Deutsches Primatenzentrum, Göttingen, Germany), as well as with the institutional guidelines of the Department of Biomedical Sciences of the University of Antwerp.

## Electrophysiological Recordings in Primates

Extracellular recordings from the oculomotor vermis in the cerebellum of non-human primates (Macaca mulatta), were performed as described earlier (Prsa et al., 2009), in three male animals of different ages, over a time span of more than a year for the purpose of other research projects. During the recordings monkeys were painlessly head fixed and the eye position was continuously monitored by scleral search coils. In four of the recordings analyzed, the monkey was instructed to actively make visually guided saccades prompted by a jumping target on a CRT monitor at 35–40 cm distance, centered in front of the monkey. In this visually guided saccade paradigm a white target dot (diameter 0.2 degrees) was presented on the monitor at the beginning of each trial. After a successful fixation period within an invisible rectangular window of ±1 degrees from the center of the dot for 500–1000 ms, the dot shifted to one of the 8 possible locations (horizontal, vertical and oblique) at a radial eccentricity of 10 degrees prompting a visually guided saccade. Each correct trial was rewarded with a unit of liquid (juice or water). Six additional recordings were obtained while the monkey was sitting in the dark without instruction or reward and executed spontaneous saccades.

Glass-coated tungsten microelectrodes (Alpha–Omega Engineering, Nazareth, Israel) with an impedance of 0.8–2 MΩ were employed to record extracellular raw voltage signals. The low impedance and fine tip of these electrodes provides low noise and excellent single cell discrimination, which was essential for this work. Signals sampled at 25 kHz and amplified 3000 times were both band-pass filtered between 300 and 3000 Hz and low pass filtered (<250 HZ). These filtering settings allowed separating spiking activity from local field potentials. Furthermore a notch filter was applied to filter out any 50 Hz noise induced by the power-line. PC activity was identified by the simultaneous occurrence of SSs and CSs and the above-mentioned characteristic pause in the SS train after a CS. Only traces showing clear CS waveforms were used for further analysis, performed off-line by custom MATLAB scripts (The Mathworks, Natick, USA).

## Complex Spikes Detection and Categorization

We only analyzed recordings with stable SSs and CSs amplitudes to ensure the stability of the experimental conditions over time, possibly affected by electrode position drifts. Traces showing periods with distinct stable amplitudes were instead split into separate segments, for further spike recognition.

**Figure 1:**
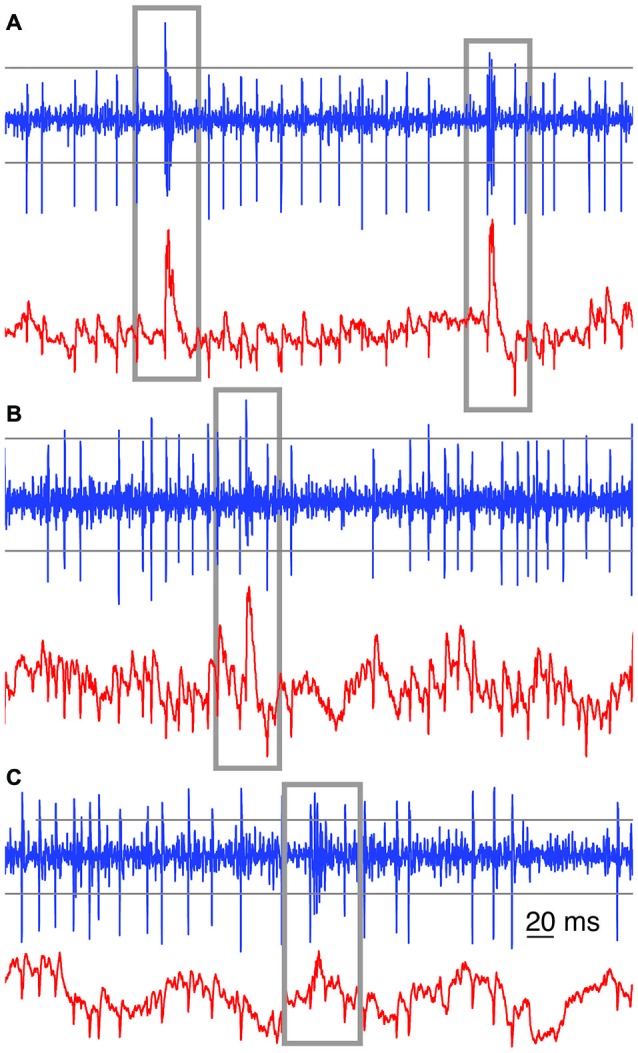
**Examples of automated complex spikes (CS) recognition**. Gray boxes were drawn around each recognized CS among the SSs, in high band-pass filtered (blue trace) and low-pass filtered signals (red trace) from three raw extracellular voltage recordings. In **(A)** the imprints of the CSs on the low (red) and band pass filtered (blue) traces are clearly visible and either of them is sufficient to detect CSs reliably. In **(B)** the imprint of the CS on the low-pass filtered trace (red) provides a better means for CS recognition. In **(C)** a combined approach was used. Closely following threshold recognition in the band-pass filtered signal would give a fairly good CS recognition. Parameters were set to prevent false-negatives. Across the panels, the horizontal gray lines depict sample thresholds for CS detection in the band-pass filtered (300–3000 Hz) signals. For the low-pass (<250 Hz) filtered signal, a sliding window threshold was used instead (not shown).

From each 300–3000 Hz band-pass filtered extracellular voltage signal, the first 20 CSs were manually selected in order to sample their shape variety. Subsequent CS selection was based on semi-automated detection, using multiple methods based on combining both high band-pass and low-pass filtered extracellular signals. The main method for CS detection is based on voltage-threshold crossing on high band-pass filtered signals, which could only be used if the CS’s first spikelet peak amplitude substantially differed from SS amplitudes (Figure 1A). The second method was based on the imprint of the CS on the low-pass filtered signal, causing an upward voltage deflection (Figures 1A,B). Due to the spontaneous slow waves in the local-field potentials, however, the threshold level for this detection method was determined with a moving average, whose length was manually chosen for each recording (12–40 ms). The third method was based on a combination of parameters describing common features of the CSs, as observed in the high band-pass filtered signal: e.g., an amplitude threshold for the first spikelet and a time window, combined with an amplitude threshold for the second or third spikelet (Figure 1C). For this last method the event was only detected as a potential CS if all mentioned criteria were met. This performed well because of the homogeneity in the initial parts of the CS waveforms, as it can be seen in the overlays of Figures 4A, 5A. The parameters of each method used were chosen in such a way that all manually selected 20 CSs were easily detected by the method. Subsequently a second by second screening of the trace was performed to verify that our automated methods did not miss CSs.

Then, the voltage samples corresponding to each of the events detected by these methods were stored in a repository of potential CSs. Each one was afterwards checked, comparing its shape on the CS characteristics, such as amplitude of the signal, number of spikelets and the similarity to the average CS waveform. In principle, two closely following SSs might be erroneously interpreted as a CS. However, such “false” CSs could easily be discarded because of the longer duration of an individual SS compared to a CS spikelet and the lack of a following SS pause.

To give an overview of the amount of variability in CS shapes, the validated repository of waveforms was further categorized into waveform groups. To this end, each CS was aligned at the point of its highest voltage increase, i.e., its largest upstroke velocity. The aligned overlay of the CSs shows a high degree of uniformity of the initial parts of the CSs (Figures 4A, 5A—black traces). Conversely, the late parts show increasing variation, both in amplitude, spikelet count and spikelet timing. An immediate striking observation, among the CSs, is the different numbers of spikelets. Moreover, we noticed that among CSs with equal number of spikelets the timing of spikelets varied. Despite the large waveform variability it was obvious that CSs from the same recording occur in distinguishable categories with similar shapes.

Different approaches to automatically divide the waveforms into separate categories were attempted. We explored the differentiation of CS duration based on energy levels in frequency bands (von Tscharner, 2000), and the differentiation based on the Mahalanobis distance (Mahalanobis, 1936). But neither of the methods provided a sufficient discrimination level. Categorizing waveforms based on mean square differences (MSD) between individual CSs and preselected waveforms did help to distinguish the CSs collected, but implied that a pre-categorization would have had to be made. Because none of the automated methods was completely reliable, the characterization of different categories of CS waveforms was done manually, guided by similarity measures (MSD).

To sort the CSs, clear waveforms showing the most distinct spikelets were selected to set possible categories *a priori*. Subsequently, the CSs were plotted one by one, superimposing them on previously categorized waveforms. A CS showing good similarity to an already categorized waveform group would then be assigned to that category, while a CS not fitting to any of the previous categories would be recognized as the first instance of a new category. Differences in the duration of CSs, the spikelet timing, and the spikelet amplitudes were taken into account. About 30–40% of all CSs in a recording were not assigned to any category in this initial round, and were grouped together.

Once all categories were determined, every CS was checked again. At that stage, CSs could be reassigned to a different category, if this improved their MSD, and most of the unassigned CSs could still be categorized. If an unassigned CS showed large MSD values to all categories, it was appointed to an “uncategorized” set, which comprised 2–10% of all CSs in a recording.

A second sorting of the CSs in each recording was made by grouping CS categories together whose CSs have an equal number of spikelets, these were named “classes” (Figure 4A). In a few recordings, CSs categories with an equal numbers of spikelets, but showing strong systematic divergence of waveforms, were grouped in different classes.

To illustrate the consistency of CS waveforms within a category, and to justify the assignment to different categories, two approaches were used. An approach based on visual inspection; comparing overlays of single CSs within a category and comparing overlays of mean waveforms of different categories. In Figures 4A, 5A, the 95% confidence interval of the CSs in each category around its mean waveform shows the waveform consistency per category, the overlay of the mean waveforms of all categories show the variability in shapes observed in a single neuron. The second approach is feature based, landmark points of single CSs were compared, such as onset times of spikelets (Figures 4B/C, 5B/C).

## Spikelet Identification, Timing and Amplitude

The identification of the spikelets is somewhat challenging, due to the heterogeneities of the maximum and minimal voltage levels in their extracellular recordings. Moreover, the spikelet voltage range changes slightly over the duration of the CS waveform. In fact, towards the end of most CSs the spikelets decline in amplitude, though some CSs show an initial decline in amplitude followed by an increasing voltage amplitude at the very end of the CS. Extracellular voltage deflections are considered spikelets only if they have maximum and minimum voltage levels relatively close to their preceding and following voltage deflection (i.e., spikelet). For instance, the waveforms of category A from recording 2 (Figure 4A) have two recognized spikelets. The small bump in between these two is not defined as a spikelet, because its maximum voltage is not in line with the spikelets around it. Spikelet times were finally determined as the time-points of their lowest voltage value (trough). Histograms of the trough time-points were fitted with a normal distribution, using MATLAB’s function. The distributions of the spikelet times were compared for consistency of difference between waveforms of different categories. A measurement of separation between categories was provided by signal-to-noise (S/N) ratios (Dayan and Willshaw, 1991; Graham, 2001), computed as

(1) $$S/N_{m}=\frac{2{\left(<Spkl_{m}^{A}>-<Spkl_{m}^{B}>\right)}^{2}}{\sigma {\left(Spkl_{m}^{A}\right)}^{2}+\sigma {\left(Spkl_{m}^{B}\right)}^{2}}$$

where

$Spkl_{m}^{A}$: Times of the trough in spikelet number *m* of category *A*;

$Spkl_{m}^{B}$: Times of the trough in spikelet number *m* of category *B*.

In the nominator we use the mean spikelet times (<…>) for two categories, in the denominator we use standard deviations (σ) of spikelet times for two categories.

## Complex Spikes Waveform Order

To test whether or not the waveform groups occurred randomly throughout the recording, we looked at waveforms of neighboring CSs. The counts of neighboring pairs of both categories and classes were checked with a bootstrap method. Histograms of neighboring pairs coming from 10000 randomized CS waveform sequences gave the upper and lower accuracy limits, rejecting a random order determined by *p* < 0.05 and *p* > 0.95 significance values.

## Inter Complex Spike Interval Lengths vs. Spikelet Jitter

Although CSs with equal number of spikelets showed strong uniformity in their waveforms, the last one or two spikelets often showed a deviation in their timing. The time distributions of the last spikelets of different CS categories (with equal number of spikelets) were compared using the S/N tests, showing significant differences in timing of spikelets at the end of the CSs. The time delay of spikelets per CS category was defined as the mean time between the spikelet of interest and its corresponding spikelet in the shortest duration CS category (Figures 6A, 7A).

Subsequently we tested the correlation between CS groups ordered on CS duration and the preceding CS interval. First linear fits were obtained by linear regression, using MATLAB’s polyfit function, on the interval lengths vs. CS duration groups, as seen in Figures 6C, 7C. The significance of the slope was then tested with a bootstrap method, by comparing it to the distribution of slopes found by shuffling the CS durations over the groups. Shuffling was done 10000 times and the significance was set at *p* < 0.05.

We further tested the correlation between the average jitter per category and preceding CS interval, seen in Figures 6D, 7D. Lastly we analyzed the duration of CSs, time between first and last spikelet, vs. preceding CS interval (Figures 6E, 7E). Data was fitted to a power function. Significance of correlation between the fitted line and data was tested using a cross correlation test.

## Saccade Detection and Related CS Time Window

Saccades were detected offline using custom written MATLAB scripts. Eye velocity was filtered using a Gaussian filter and thresholded to find saccade onsets and offsets. The threshold was based on the mean velocity of the eyes during the recording and a scaling factor times the standard deviation of the velocity. All CSs within a window of 100 ms before and 175 ms after saccade-offset (Catz et al., 2005; Soetedjo et al., 2008) were considered to be potentially saccade related.

## Complex Spikes—Simple Spikes Pause Lengths

CSs triggered prominent pauses in the SS trains. We looked whether different CS classes in a PC trigger SS pauses of different durations, reported with ± standard error of the mean (SEM). These durations were obtained by evaluating the time from the onset of the CS to the first following SS, which were detected by threshold crossing. We took the CS onset rather than its end, because of its higher signal-to-noise ratio.

### Complex Spikes Triggered Simple Spike Frequency Change

CSs can trigger plasticity mechanisms in the PC and thus have a potential role in modulating the spiking activity of the PC. We obtained SS activity in time windows ranging from 150 ms to 500 ms, both before and after each CS. The window before the CS is directly preceding the onset of the CSs. The window used for the post CS SS activity starts at a time point where 90% of all CS triggered SS pause lengths ended. For multiple time windows per PC we looked at CS waveforms and their SS firing rates either before or after the CSs. As the optimal time window in which SS firing rates change is unknown we analyzed each time window comparison individually.

### *In Vitro* Electrophysiological Recordings in Brain Slices

We performed *in vitro* recordings from PCs on cerebellar slices, in total 18 neurons were recorded from acute cerebellar tissue obtained from 12 animals. Wistar rats (P18–26) of either sex were anesthetized with 4% isoflurane and decapitated, sagittal slices (300 μm) of the cerebellar vermis were prepared by standard methods. Briefly, slices were cut in an ice-cold low calcium and high magnesium solution, containing (in mM) 125 NaCl, 25 NaHCO_3_, 25 Glu, 2.5 KCl, 1.25 NaH_2_PO_4_, 1 CaCl_2_, 4 MgCl_2_, using a vibratome tissue slicer (VT 1000 S, Leica Microsystems, Germany). Slices were incubated at 32°C in a standard artificial cerebrospinal fluid (ACSF), containing (in mM) 125 NaCl, 25 NaHCO_3_, 25 Glu, 2.5 KCl, 1.25 NaH_2_PO_4_, 2 CaCl_2_, 1 MgCl_2_, for 30–45 min and then somatic whole cell patch-clamp recordings were performed at 34 ± 1°C using 3–6 MΩ borosilicate glass pipettes, filled with a solution containing (in mM) 130 KMeSO_4_, 7 KCl, 10 HEPES, 0.05 EGTA, 2 Na_2_ATP, 2 MgATP, 0.5 Na_2_GTP (adjusted to a pH of 7.3).

Current-clamp recordings were obtained using a Multiclamp 700B amplifier (Molecular Devices, California, USA), low-pass filtering voltage traces at 10 kHz, and sampling at 30 kHz, with the LCG software (Linaro et al., 2014). Pipette capacitance and resistance were carefully compensated during the experiment by the amplifiers circuitry. All recordings were performed in the presence of SR95531 (GABA_A_ receptors blocker, Sigma-Aldrich, Belgium).

A theta glass pipette, filled with ACSF, was used to deliver bipolar, monophasic cathodic electrical extracellular stimuli (0.2 ms, 150–500 mV) to the granule cell layer. Care was taken to isolate the CF responses and to employ minimal stimulation amplitudes. In some cases, pulses separated by 2–3 ms, were employed, aimed at mimicking IO bursts (as in Mathy et al., 2009). Two different stimulation protocols were used. The first consisted of blocks with constant intervals of 0.2, 0.4, 1.0, 2.0 or 5.0 s. This protocol was used for 11 neurons from 8 different animals. In the second protocol, used in 7 neurons from 4 different animals, the intervals were randomly drawn from the normal distribution. Complex spike waveforms were identified in intracellular voltage recordings, and spikelet detection and further analysis performed by custom MATLAB scripts.

The CF stimulation protocols mostly triggered CS with equal spikelet numbers; the few CSs with more or less spikelets were discarded for the purpose of a fair comparison of CS duration. To test the correlation between CS duration and the preceding CF stimulation interval in the *in vitro* data we analyzed the linear fit, using MATLAB’s polyfit routine, of the data seen in Figures 8B,C, 9B. The significance of the slopes was tested using a bootstrapping method, as for the *in vivo* data. The CS durations and the preceding CF stimulation interval pairs of a recording session were shuffled and a linear fit value was found. This was done 10000 times giving a distribution of linear fits from shuffled trials. Significance was reached when the empirical found slope was in the 5% border values of the shuffled linear fit distribution (*p* < 0.05).

## Results

**Figure 2:**
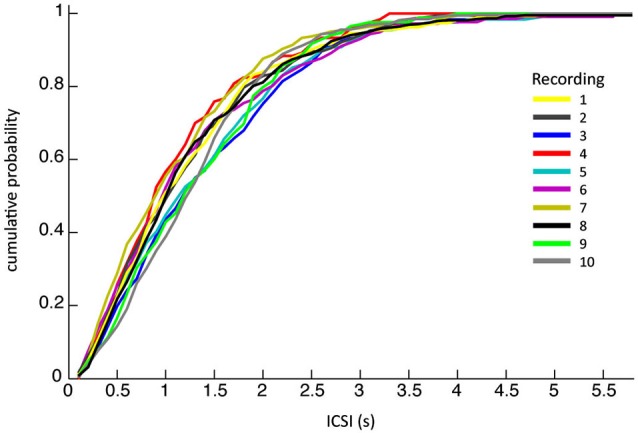
**The cumulative probability of inter complex spike interval (ICSI) lengths**. The ICSI distributions of the ten different recordings across different monkeys on different days show remarkable similarity.

The ten PC recordings from awake behaving primates analyzed had mean durations of ~6 min each (see Table 1 for detailed overview). A total of 2789 CSs were detected, occurring at an average frequency of 0.8 ± 0.8 Hz (SEM), the corresponding inter complex spike intervals (ICSI) were on average 1254 ± 910 ms, similar to previously reported CS firing rates (Armstrong and Rawson, 1979). Their high variability stands out even though cumulative distributions of ICSIs were similar for all PCs (Figure 2), suggesting that all recordings were obtained under comparable conditions.

**Table 1:** **An overview of 10 cells of which the CSs were categorized spanning over 1 h of data**.

Recording	Recording length (s)	Mean ICSI (ms)	SEM (ms)	# of spikelets	Length time (ms)	# of CSs	# of categories	Condition
1	322	1231	±940	2–5	4–9	263	12	target sac
2	386	1220	±900	2–5	4–10	316	10	target sac
3	651	1390	±975	2–4	7–9	469	15	target sac
4	133	1101	±798	4–6	7–10	121	13	dark
5	321	1353	±994	3–6	6–11	237	17	dark
6	268	1260	±1031	6–10	7–10	213	16	dark
7	331	1108	±905	6–10	7–10	300	20	dark
8	465	1259	±938	2–6	3–10	386	16	target sac
9	225	1322	±829	4–7	6–9	169	9	dark
10	408	1299	±791	4–7	4–8	315	26	dark
Mean all Recordings	351	1254	910	3.4–6.5	5–10	279	15
SEM	138	135	143			97	5
Total	>1 h					2789

*Recordings on average were 351 s long. In total 2789 CSs were detected and categorized, the frequency of occurrence on average is 0.8 Hz. A noticeable feature is that where the average frequency over all the recordings is very consistent, 0.8 ± 0.08 Hz (SEM) the intervals between CSs in single recordings show very large fluctuations resulting in large SEM values for each recording. The number of spikelets gives a general impression of the variation of CS waveforms found. CSs durations are based on the time between the onset of the first and end of the last recognized spikelet. The “# of categories” column indicates the number of CS categories found in each recording. The last column indicates the experimental condition during which the recording was obtained, which is either the monkey being instructed through targets appearing on a screen to make visually guided saccades (target sac) or is sitting in the dark without any saccade instructions (dark)*.

### Waveform Variability

**Figure 3:**
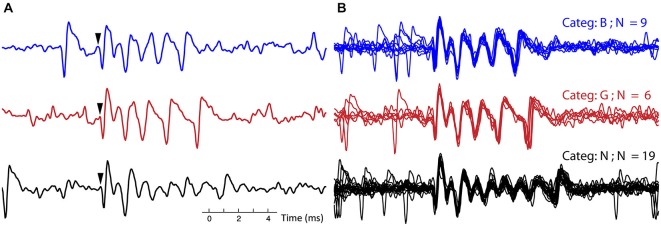
**Example of complex spike categories. (A)** Three single CSs from recording 7, each CS belongs to a different category, in both the blue (top) trace and black (bottom) trace a simple spike (SS) precedes the CSs. Arrows indicate onset of the CS. **(B)** All the CSs belonging to each of the three categories, corresponding to the ones in **(A)**, are overlaid for this recording. The CSs from the three different categories (out of 10 total for this recording) were selected to exemplify clear differences between categories. (Categ. = category).

Complex spikes variability has been reported in single cells *in vitro* (Khaliq and Raman, 2005; Tal et al., 2008), here we describe the CS variability *in vivo* in a systematic manner. For each recording, CSs were classified into categories, based on the similarity of their waveforms (see Section Methods). Figure 3 compares single traces from three representative CS categories within a single recording to highlight the differences. The initial parts of the waveforms over the three CS categories (~2 ms) show high similarity, but the overall CS duration and the number of spikelets differed greatly (Figure 3A). Figure 3B shows the variability within each CS category: the CS duration and spikelet number are identical but individual CS traces are noisy and do not overlap perfectly.

**Figure 4:**
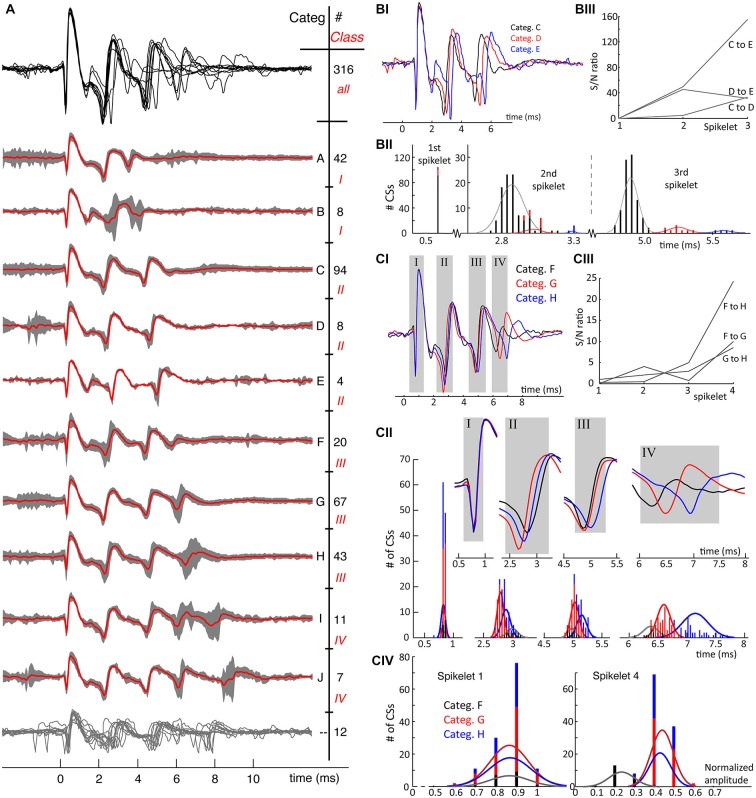
**(A)** From recording 2, the mean waveforms of each CS category are plotted overlaid in the upper trace in black. A clear observation is the homogeneity of the first spikelets over all categories and the increasing waveform variability towards the end of the spike. Thereunder the mean waveforms of each category are plotted in red, overlying the 95% confidence interval of the CSs in each category in gray. CS categories are plotted orderly according to duration of the CS. Each category is indicated with a letter on the right and the number of single CSs belonging to it is also given. A second distinction among the CSs categories was made based on the number of spikelets present: this classification shows 4 different classes, indicated with roman numbers, and shows an increase of spikelet count going from 2 till 5. Twelve CSs out of 316 could not be assigned to any category, the twelve individual CS traces are overlaid in the bottom having no category letter. **(BI)** Overlay of three mean waveforms of the comparable but distinguishable CS categories, C, D and E all having 3 spikelets. The first spikelet is identical in all three mean waveforms, with the strongest difference being in the timing of the third spikelet. **(BII)** The distribution of the spikelet timings over all CSs in categories C, D and E. While these distributions perfectly overlap for the first spikelet, they are completely separated for the third one. Normal distribution profiles were fitted to the second and third spikelet timings for each category. Notice the large difference in counts, due to unequal category sizes. **(BIII)** The signal-to-noise (S/N) ratio’s of the spikelet timing between the different categories confirm the spread of the spikelet times towards the end of the CSs. **(CI)** CS mean waveforms of the categories F, G and H, class III, differ in timing and amplitudes of last spikelets across categories. **(CII)** Full length CS mean waveforms of the three categories being compared, the gray rectangles correspond to the rectangles in **(CI)**. The upper panels show the blown up parts of the mean CS waveforms used to determine the timing of each spikelet. Below are the histograms of the timing of the four spikelets of individual CSs per category. Normal distributions fitting the time population per category for each spikelet are superimposed. These show a clear spread of timing for the fourth spikelet, again confirmed by the S/N ratio’s shown in **(CIII). (CIV)** Maximal amplitudes of the 1st and 4th spikelet of every single CS for each category. While these distributions again overlap for the first spikelet, category F shows clear amplitude differences of its last spikelet compared to G and H. D. The signal-to-noise ratios of the spikelet time populations of category F, G and H. (Categ. = category).

**Figure 5:**
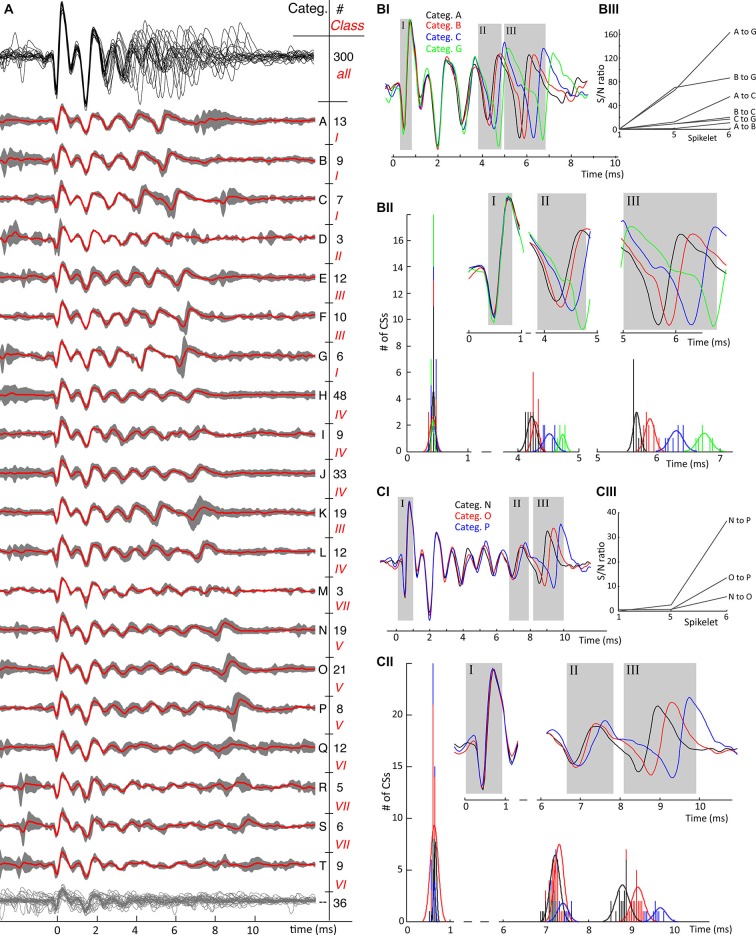
**Second example of mean category waveforms and their variability. (A)** An overview of identified waveform categories from cell 7, same conventions as in Figure 4A. The waveforms of all categories show a uniform initial phase as shown by the upper waveform overlay in black where after the number of spikelets, their timing and their amplitudes differ. The CSs in this cell show 7 classes, ranging from 6 clear spikelets, class I, till 10 spikelets in class VII, which are harder to distinguish although not impossible. Categories Q, R, S and T have low amplitude late spikelets and therefore have less characteristic features. In this recording 36 CSs out of 300 could not be categorized, grouped together at the bottom having no category letter. **(BI)** Timing of last spikelet(s) shows jitter between CS categories. A similar convention as in Figures 4CI–III is used: The full-length mean waveforms compared are shown in the **(B/C–I)** block. Spikelets further analyzed are shown in the gray rectangles in the **(B/C–II)** blocks corresponding with the ones in the mean waveform panels. The time histograms in each comparison show the time distributions of the analyzedare spikelets. The signal-to-noise ratios between the spikelet time populations shown in the **(B/C–III)** blocks. The S/N ratios in both comparisons demonstrate the spread of spikelet timing towards the end of the CSs. B. Comparison of categories with 6 spikelets. The timings of both the second-to-last (categories A and B vs. C and G) and last spikelet (all categories) do not overlap. C. Comparison of categories with 9 spikelets. For these categories only the last spikelet is well separated. (Categ. = category).

The examples shown in Figure 3 are only a subset of a much larger variety of categories observed in this neuron. On average, 15 ± 5 different categories were observed in each PC recording (Table 1). Complete overviews of all categories from recordings of two different neurons are shown in Figures 4A, 5A. The different categories found in a single PC were sorted by increasing duration, and for convenience labeled by a letter. The number of CSs in a category varied from 1 to 30% of all CSs in the recording and each category occurred throughout the entire duration of each recording. The differences of CS waveforms between recordings from different PCs were, in general, larger than the differences within a single recording. As exemplified in Figures 4, 5 this was mainly due to large differences in the total duration of the CS between different PCs.

Figures 4A, 5A show the consistency of waveforms within each category: the mean waveform, in red, overlays the category 95% confidence interval. The top of the figures shows an overlay of all mean waveforms, demonstrating that waveform variability increases towards the end of CSs across categories. From the confidence interval plots a similar variability can be seen within categories.

A conspicuous characteristic of the CS categories is their spikelet count, which defines a secondary grouping, named “classes”. e.g., in recording 2, the number of spikelets per CS ranged from 2 to 5 (Figure 4A). In a few exceptional cases, CS categories with equal spikelet numbers were recognized as different classes, because of large differences in amplitude and spikelet timing. Classes II and III, recording 7, exemplify these cases (Figure 5A). Some CS categories have a rather unique shape while others show strong common similarities.

**Figure 6:**
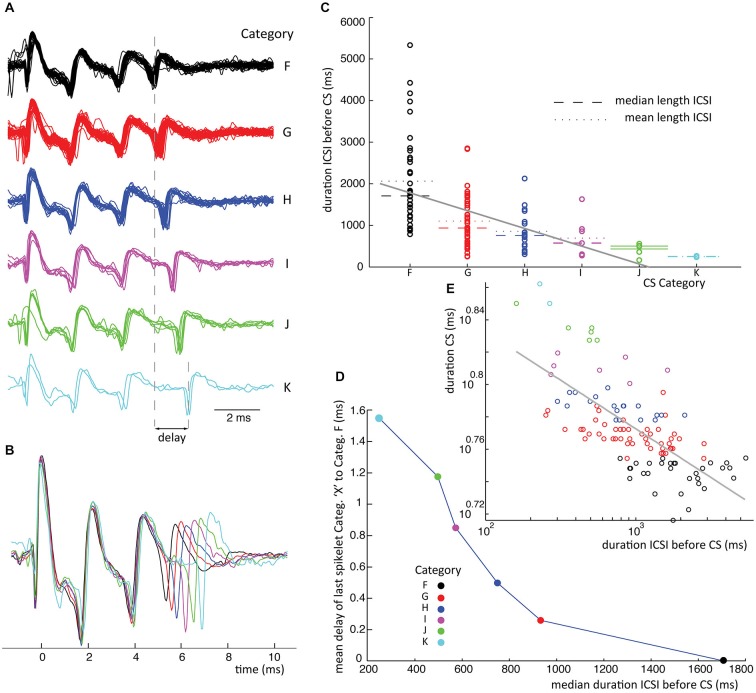
**ICSI duration before CS sets timing of last spikelet in CSs. (A)** Waveforms from class III in recording 1 for which delay of last spikelet and total duration has been measured. **(B)** Superimposed mean waveforms of each category. **(C)** ICSI times preceding all class III CSs (recording 1) are plotted separated on category. The spread of the ICSI times is large for the different categories; of each the mean (stippled line) and median (broken line) time is given. A linear fit of the data, gray line, shows a significant declining slope on bootstrap testing (*p* < 0.05). **(D)** Median ICSI lengths are plotted against the average delay of the last spikelet per category. The delay is obtained by taking the average time difference between the last spikelet of each category and the last spikelet of the shortest CS (category F), as shown in panel **(A)**. The figure shows the shorter the median time length of the ICSI before the CS, the longer the mean delay of the last spikelet. **(E)** Scatterplot of the time between first and last spikelet vs. ICSI times before CSs in a log-log plot. Gray line is a power-function fit (power = −0.026) to the data with *R*^2^ = 0.49. Correlation between data and power-function is significant (*p* < 0.001). Colors used indicate categories. (ICSI = inter complex spike interval, Categ. = category).

Waveform shape differences among CS categories from recording 2 (Figure 4) are analyzed in detail in Figures 4B,C while waveform variability in recording 7 (Figure 5) is shown in Figures 5B,C. For a third recording, (recording 1) waveforms from class III are shown in Figure 6. Recognized CS classes and their characteristics for all 10 recordings are summarized in Table 2. For the counts of CSs grouped on spikelet number per neuron, there was a trend that CSs with a spikelet number closer to the neuron’s average, appear more frequently (Table 2).

**Table 2:** **The classes (Cl) of CSs, based on number of spikelets (spklt) for all recordings**.

Recording	Cl I # CSs	# spklt	Cl II # CSs	# spklt	Cl III # CSs	# spklt	Cl IV # CSs	# spklt	Cl V # CSs	# spklt	Cl VI # CSs	# spklt	Cl VII #CSs	# spklt
1	40	2	95	3	121	4	3	5
2	50	2	106	3	130	4	18	5
3	29	2	54	3	79	3	212	3	94	4
4	46	4	2	5	4	5	5	5	35	5	2	6
5	34	3	30	4	25	5	57	5	63	4	4	5	10	6
6	14	6	29	7	80	8	42	9	4	9	7	10
7	35	6	3	7	41	7	102	8	48	9	21	10	14	10
8	12	2	136	3	88	3	90	4	51	5	4	6
9	10	4	27	5	116	6	12	7
10	10	4	66	5	213	6	13	7	13	7

*For each class the number of CSs and the number of spikelets is shown. Notice that CS classes with an intermediate number of spikelets tend to be larger than the classes with lower or higher spikelet numbers*.

### Timing of the Last Spikelet is Most Variable

In Figure 4B we compare the mean waveforms of all categories from recording 2 with three spikelets. The waveform overlay shows that the first spikelet is identical and the shapes of the following spikelets are very similar among the three categories. The main difference is the timing of the third and, to a lesser degree, the second spikelet. Timing differences are summarized in Figure 4BII: the time distributions of the last spikelet are completely separated across the categories, time distributions of the second spikelet show partial overlap for categories C and D but are separated for category E. High S/N ratios (Figure 4BIII) of the last spikelet time distributions between categories demonstrate the increased spread towards the CS end.

In Figure 4C, we compare the mean waveform of the categories with 4 spikelets, categories F, G and H of recording 2. We observe again that the timing of the last spikelet differs between the categories (Figure 4CII). Although the separation in this comparison is less defined, the S/N ratios highlight a clear difference (Figure 4CIII). A more distinct difference between categories F and G is the amplitude of the last spikelet (Figure 4CIV): the amplitude of the fourth spikelet is comparable between category G and H, but is much smaller for category F.

Additional examples of the consistent variability of CS shapes are presented for a second recording, cell 7, in Figure 5. The CSs in this PC last significantly longer than those in the previous example and comprise 6–10 spikelets. Categories R, S and T had consistently smaller amplitude spikelets that made them harder to distinguish. As already observed, the initial segment of the CS waveform, here comprising the first 3 spikelets, is similarly shaped in all categories, while the variety between categories increases toward the CS end. In Figures 5B,C, two waveform shape comparisons of CSs with equal number of spikelets show the variability in timing of the last spikelets among categories. Categories A, B, C and G in Figure 5B show significant jitter (among each other) in both the second-to-last and last spikelet. Categories N, O and P in Figure 5C show instead variability only for the last spikelet.

A complete overview of the variation of CS waveforms per recording, with respect to the number of spikelets and the duration of the CSs is shown in Table 1. Profound differences were found in the spikelet count range among CSs from different cells (2–4 vs. 6–10). Between categories with the same number of spikelets, the timing of the last spikelet was the most significant difference. Over all 10 cells recorded, this finding was consistent for 9 cells. In 8 out of these 9 cells, the timing of the second-to-last spikelet also varied among categories, although to a lesser extent. The timing of the earlier spikelets was consistent between categories from a single class. Only few CS categories showed amplitude differences (Figure 4CIV), but these were always accompanied by large S/N ratios for the time shifts.

### No Sequence of Occurrence of Different Complex Spike Shapes

The occurrence of CS waveforms, both classes and categories, do not show any consistent sequence throughout the recording. Using bootstrap methods, we tested the significance of pairs of categories following each other in the same recordings. No single recording showed a preference in the category order. The same method also excluded a pair wise order preference for CS classes.

We compared the range of CS shapes observed during the two different experimental conditions. Four recordings were obtained when monkeys were making visually guided saccades for rewards and six recordings were obtained when the monkey was sitting in the dark without any instructions. Both conditions showed comparable ranges of CS shapes with respect to the number of spikelets and the length of the CS, see Table 1. Also there were no differences between the conditions in the number of CS classes (Table 2, *p* > 0.67 two sided KS test) or number of categories (Table 1, *p* > 0.44). Next we investigated whether CS related to saccades (occurring in a time window from 100 ms before to 175 ms after the saccade offset) had different shapes compared to CSs outside these time windows. In all the 10 recordings the distributions of CS classes, saccade related vs. saccade unrelated, appeared random.

### Complex Spike Shapes Relate to the Duration of the Preceding Interval

We investigated possible relations between the presence of specific CS waveform categories and other features of the PC spike train: SS pauses, SS rate and CS rate.

A well-known feature of the CS is that it induces a pause in the SS train (Bell and Grimm, 1969; Latham and Paul, 1971; McDevitt et al., 1982). Moreover dendritic Ca^2+^ spikes during the CS were found to regulate the afterhyperpolarization amplitudes and therefore modulate post CS SS-pause lengths (Davie et al., 2008). The pause duration distributions over the ten PC recordings showed differences. The average of all the mean pause durations from the 10 recordings was 26.1 ± 6.6 ms (SEM). But neither the waveform ranges over the different PCs seemed to correlate to that cell’s mean pause duration nor did the CS categories or classes in single neurons correlate to the pause duration.

Next we investigated possible correlations between the SS rate, preceding or following a CS and its category. Because of the highly fluctuating interspike intervals we looked at different time ranges (i.e., 150–500 ms), before and after the CS. The CS waveforms did not depend on the preceding SS rate and they did not induce repeatable changes in SS rate, neither in the relative nor in the absolute rate change.

**Figure 7:**
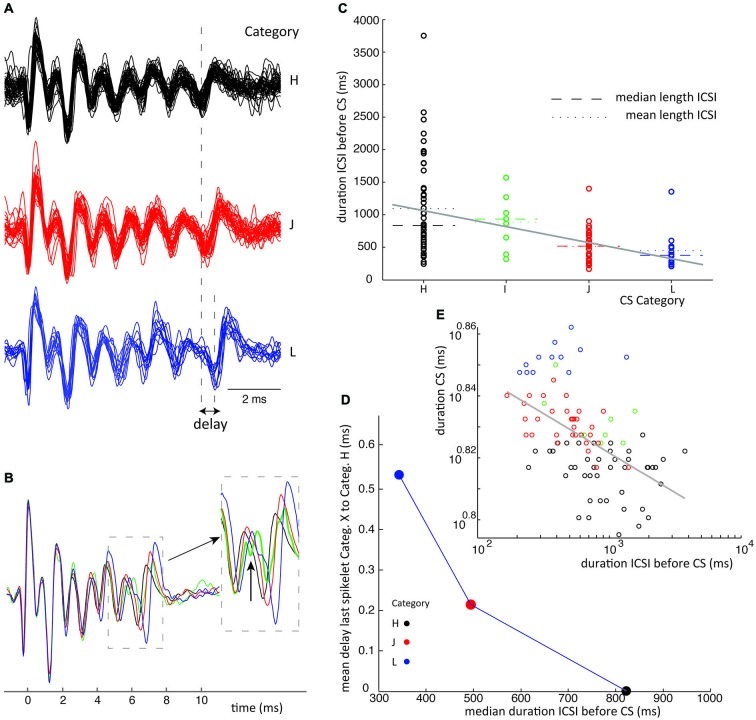
**ICSI duration before CS sets timing of last spikelet in CSs.** Similar conventions are used as in Figure 6. This is an example which does not perfectly show the ICSI time vs. spikelet jitter phenomena as stated in Figure 6 because category I has a longer ICSI than category H. However category I shows an odd shape of its fore last spikelet as shown in panel **(B)** and might belong to a different class of CSs. **(D)** Leaving category I out we do find a consistent relation between ICSI and last spikelet delay. **(E)** Power function fit had power of −0.06, *R*^2^ = 0.3, *p* < 0.001 for the correlation between fitted function and data.

Finally we investigated the relation between CS categories in a single class and the ICSI, both before and after the CS. The scatter plot in Figure 6C shows the distribution of ICSIs before CSs of 6 categories belonging to the same class. The CS waveforms differ only in the jitter of their last spikelets, as can be seen in Figures 6A,B. The ICSI lengths per category show a large spread (Figure 6C), but the median ICSI length before the CSs of different categories shows a consistent correlation with the last spikelet timing (Figure 6D). The spread between the first and last spikelet grows as the ICSI before the CS gets shorter. In total, we looked at the 28 biggest classes of all ten recordings, from which 19 consistently showed an inverse correlation between the timing of the last spikelet and the preceding ICSI, without exceptions (Figure 6C). This relation was statistically significant at *p* < 0.05 in 15 of the 19 data sets, in the other cases the data sets were too small to reach significance. In six classes we saw a similar correlation but with a single outlier, an example is given in Figure 7, and in three classes the correlation was not found. CS shapes did not correlate to the directly following ICSI length. These results confirm that CSs within a class are comparable and differ mostly in the jitter of the last spikelet(s), which is determined by the preceding ICSI. To exclude that our observations were a consequence of the manual categorization of CS waveforms we repeated this analysis by looking at the dependence of the duration of the CS, measured as the distance between first and last spikelets, on preceding ICSI and confirmed the findings (Figures 6E, 7E).

The consistent ICSI-jitter correlation was found in CS classes having 2–9 spikelets and covered the full range of the recordings. The three classes not consistent with the finding were also randomly distributed over the observed spikelet number range.

### Complex Spike Shape Changes *in Vitro* Confirm *in Vivo* Findings

We hypothesized that the effect of the ICSI length on timing of the last CS spikelet could arise from ICSI dependent changes in PC excitability. If this were true, similar findings would occur for CSs evoked by CF stimulation in an *in vitro* slice preparation, without influence of the IO. We therefore carried out patch-clamp recordings of PCs in rat cerebellar slices, and evoked CSs by extracellular electrical stimulation of the CF (see Section Methods). This allowed us to artificially modify the ICSI in a consistent manner, by changing the frequency of CF stimulation. We could sometimes evoke long CSs waveforms with many spikelets, however since these were non-reproducible over long timescales, we restricted the analysis to short CS waveforms (<15 ms).

**Figure 8:**
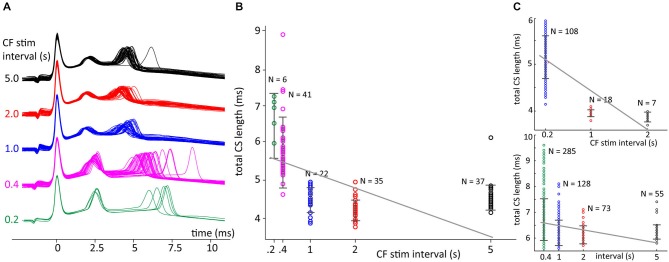
**Climbing fiber (CF) stimulation interval sets delay of the last spikelet. (A)** Patch clamp recordings of CF stimulation triggered CSs in cerebellar slices with CF stimulus intervals of 0.2, 0.4, 1.0, 2.0 or 5.0 s. **(B)** Total duration CS, time between first and last spikelet peak, sorted on preceding CF trigger interval length. Error bars indicate standard deviation. A linear fit of the data, gray line, was found to have a significant declining slope (*p* < 0.05) in bootstrap testing. **(C)** Two other recordings showing a decline in total CS duration with longer preceding CF interval times, similar convention as in panel **(B)**. Both examples also show a significant declining slope of the linear fits of the data. Notice that total CS duration doesn’t decrease anymore for an interval length increasing from 2 to 5 s, the latter interval length is not observed in the *in vivo* recordings.

In Figure 8 data is shown for CSs evoked with repeated stimulation at five different preceding ISI durations, i.e., 0.2, 0.4, 1.0, 2.0 and 5.0 s. These CSs had three spikelets and both the second and third increased their delay with decreasing ICSI, but the effect was much more pronounced for the last spikelet (Figure 8A). As shown in Figure 8B, the ICSI predicted the timing of the last spikelet (here measured as total CS duration) in a similar manner as observed in the *in vivo* recordings. Shorter preceding CF stimuli intervals also resulted in an increased variability of the timing of the last spikelet.

To test significance we used only complex spike intervals data below 2.5 s so that we could better compare with the *in vivo* experiments. Comparing the trends found between the CS durations and the fixed CF stimulation interval before the spike we found a significant increase of CS duration with shorter intervals in 13 out 15 experiments. In two slice experiments a neuron was used multiple times so that the 15 experiments were obtained from 11 different cells. The two nonsignificant results were found in the two multiple used neurons.

**Figure 9:**
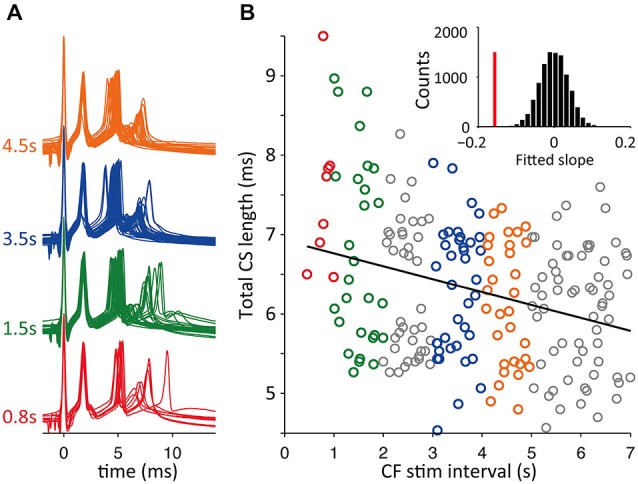
**Random CF stimulation intervals resembling *in vivo* distributions. (A)** Voltage traces of CF stimulation triggered CSs in cerebellar slices with CF stimulus intervals drawn from a normal distribution. Examples from 1 s binned intervals. **(B)** Total duration CS, time between first and last spikelet peak, sorted on preceding CF trigger interval length (as in Figure 8). Shown is the linear fit of all data points (black line) with a significant declining slope in bootstrap testing (*p* < 0.05), see inset, vertical red bar is the slope of data and the bootstrap slope distribution is shown in black bars.

A potential caveat of experiments using a fixed inter-CS interval is the presence of a built up of the adaptation of CS duration during the continued stimulation protocol, especially for the shorter intervals of 0.2 and 0.4 s. This is reflected in Figure 8 seen in the larger variance after higher stimulation frequencies. Because the ICSI duration *in vivo* shows large fluctuations such a stimulation protocol may not reflect the *in vivo* situation. We therefore repeated the slice experiments with a second stimulation protocol where the CF stimulus intervals were randomly drawn from a normal distribution and then afterwards ordered for analysis. An example of the results is shown in Figure 9. For the experiments with random interval stimulation significant correlation between CS duration and preceding ICSI at *p* < 0.05 was found in 6 out of 7 experiments (7 cells), both for the intervals below 2.5 s as for the full range of intervals (0.2–7 s) tested (Figure 9B). Based on the results shown in Figures 8, 9 we conclude that the correlation between CS duration and CF stimulus interval observed *in vivo* is also found *in vitro*.

## Discussion

### Summary of the Findings

This study shows the extent of CS waveform variability in extracellular PC recordings from awake behaving non-human primates. CSs from a single PC differed in amplitude, timing and number of spikelets. Strong homogeneity was found in the initial CS shapes (Figures 4, 5). A profound difference between CSs from different PCs was the spikelet number range. The lowest CS spikelet number observed in neuron 1 was 2 while it was 6 in neuron 7, spanning 4 and 7 ms respectively. The maximum spikelet count in these neurons was 5 and 10 respectively, spanning 9 and 10 ms (Table 1). An important difference between CSs categories in single neurons was the relative timing of their last spikelets (Figures 4B/C, 5B/C). Waveforms did not correlate to different experimental conditions during the recording. Furthermore waveforms did not show an order preference during the recording. Nor was there a correlation of waveforms with SS pause duration or with the preceding and following SS rates. The jitter of the last spikelet(s) correlated strongly with the preceding ICSI length and a similar effect was observed in *in vitro* slice studies.

### Limitations of the Study

Extracellular recordings can reveal details of intracellular spiking activity (Henze et al., 2000). However extracellular recording method limits the faithfulness and the details of the detected waveforms and many traces, containing both SSs and CSs, could not be used for categorization. Because of the explorative approach of waveform categorization, we relied on manual methods and this may lead to incorrectly categorized CSs. However, both the global analysis of waveform shape (Figures 4A, 5A) and detailed analyses of spikelet properties, combined with significant statistical tests, argue against any systematic errors.

The use of MSD guided categorization of waveforms supported the visual classification and grouped small subsets of CSs having the strongest resemblance to pre-categorized CSs (see Section Methods). Fully automated methods failed because of global waveform differences from cell to cell and insufficient shape-detail levels. There are no automated CS categorization methods described in literature at present.

### Mechanisms Underlying CS Spikelet Number

The most prominent difference between CSs is their spikelet number, found in *in vitro* recordings to relate to the number of spikes in the CF stimulation that mimics bursts of CF action potentials (Mathy et al., 2009). In the same study the CF burst size was found to correlate with the sub-threshold oscillation phase in the IO. Other authors have proposed instead that the amplitude of sub-threshold oscillations sets the number of spikes in the CF burst (Bazzigaluppi et al., 2012) and that this amplitude may be controlled by the coupling of IO cells (De Gruijl et al., 2012). Based on the absence of any preferred order of CS classes in this study, we predict that there is also no preferred order in CF burst sizes in the IO. This implies that CF burst sizes vary rapidly *in vivo*.

In between different PCs we observed profound differences in spikelet number. A possible explanation are the different physiological properties found over CFs. CFs innervating PCs in zebrin-II positive zones release more glutamate per action potential then their counterparts in zebrine negative zones leading to longer duration CS with a greater number of spikelets in zebrine positive PCs (Paukert et al., 2010). The oculomotor vermis (VI and VII lobules) in Macaca Mulatta shows clear alternating parasagittal zebrin-II expression stripes (Sillitoe et al., 2004) and it is most likely that the PCs recorded came from both types of zones.

### Mechanisms Underlying CS Spikelet Jitter

A significant part of the CS waveform variation in single cells *in vivo* was related to spikelet jitter, mainly observed in the last spikelet, sometimes in the two last spikelets. This jitter neither depended on the number of spikelets in the CS nor on the CS duration. For example, Figure 5 shows comparable spikelet jitter between CS classes having either 6 or 9 spikelets, with delays from the CS onset of 5.5 ms and 8.5 ms.

The main finding of this study is that this jitter of the last spikelet consistently depended on the interval with the preceding CS, with shorter ICSIs resulting in a longer delay of the last spikelet (Figures 6, 7). One can hypothesize two different mechanisms causing such an effect of ICSI-length on spikelet timing. Because the IO sets the ICSI, the last spikelet timing could reflect the timing of the last spike in a CF burst signal, which would then be delayed for short ICSIs (Mathy et al., 2009). Alternatively, the Olive generated ICSI could affect the excitability of the PC, most likely through the slow decay of dendritic calcium transients and accompanying differences in activation of calcium-activated K^+^ channels (Schmidt et al., 2003; Anwar et al., 2012, 2013). The central role of PC excitability in setting the timing of the last spikelets was confirmed by the slice experiments (Figures 8, 9). This strongly suggests that the jitter of the last spikelet, observed in most CSs, is due to a form of refractoriness intrinsic to the PC that underlies its delay, excluding a CF signal effect.

An additional mechanism could be the plasticity of the CF-PC synapses, since tetanization of the CF induces long-term depression of the CS (Hansel and Linden, 2000). The reduced excitatory postsynaptic potentials (EPSPs) can cause changes in the CS slow wave components in the dendrites, the depolarization plateau following the first big peak (Weber et al., 2003).

### No Other Effects of CS Shape

While we extensively investigated the relation of other parameters to CS shape and CS rate, no significant relationships could be found.

A study by Maruta et al. (2007) showed that longer preceding ICSIs correlated with a higher number of EPSP components in the CF triggered compound. Our lack of finding a correlation between CS class and preceding ICSI duration is in line with the weak correlation found between CF EPSPs and CS spikelet number in slice experiments (Davie et al., 2008; Mathy et al., 2009). Furthermore our results do not reproduce the observed paired-pulse depression of the pre-synaptic terminal of the CF-PC connection, which has been reported to results in a decreased number of spikelets of the second CS for ICSIs below 1000 ms (Hashimoto and Kano, 1998). Such differences may be due to differences in animal species or, more likely, extracellular vs. intracellular recording methods. However our findings also disagree with extracellular recordings in mice by Servais et al. (2004) which showed that the amplitude of the secondary spikelets was inversely correlated with the previous SS frequency. No influence on CS waveforms was found in our recordings, neither on the spikelet jitter nor on the number of spikelets.

We did not try to correlate CS waveforms with learning (Yang and Lisberger, 2014) because of the small number of learning trials in the analyzed data.

### Effects of CS Waveform Differences

The functional downstream relevance of different spikelet numbers in the CSs is probably limited, due to the poor propagation of somatic spikelets to the deep cerebellar nuclei (DCN). On average only 2 spikelets per CS get relayed to their downstream targets, likely indistinguishable from 2 closely following SSs (Khaliq and Raman, 2005; Monsivais et al., 2005). Moreover PC spikes induce very small inhibitory postsynaptic potentials in DCNs, suggesting negligible effect from extra spikelets (Bengtsson et al., 2011).

Waveform differences could however affect plasticity of PF-PC synapses. Single spikelet CF bursts were reported to induce long-term potentiation instead of depression (Mathy et al., 2009). Moreover in a study by Rasmussen et al. (2013) single spikelet CF bursts resulted in extinction of a learned pause response, while multiple spikelets restored the response.

CSs induce pauses in the following SS trains (Bell and Grimm, 1969; Latham and Paul, 1971; McDevitt et al., 1982), and its duration regulates the rebound effect in DCN (Aizenman and Linden, 1999). The variability in pause duration across 10 PCs was 26.1 ± 6.2 ms (SEM), falling in the 10–30 ms range reported by Shin and De Schutter (2006). Davie et al. (2008) showed the pause length to depend on the calcium spikes number in the PC dendrites. In our monkey data however, the SS pause lengths did not change for different CS waveforms, neither for different durations nor for different spikelet numbers.

Different waveforms also did not reset PC activity state differentially, based on the lack of reproducible SS rates following CS groups. This supports the finding that the presumed PC bistability triggering capacity of the CS (Loewenstein et al., 2005) is an artificial phenomena induced by anesthetics (Schonewille et al., 2006).

## Conclusion

CS waveform variability is a shared feature over many species. It has been described at different levels of detail in cats (Campbell and Hesslow, 1986) and mice (Servais et al., 2004). This study shows the surprisingly great extent of that variability in non-human primates. The ICSI length preceding the CS was found to strongly influence the CS waveform by changing the jitter of the CS last spikelets. The consistent effects of CF stimulus intervals on CS duration in slice experiments confirm that this spikelet jitter delay depends on PC intrinsic mechanisms.

## Conflict of Interest Statement

The authors declare that the research was conducted in the absence of any commercial or financial relationships that could be construed as a potential conflict of interest.
